# A selective degeneration of cholinergic neurons mediated by NRADD in an Alzheimer's disease mouse model

**DOI:** 10.1016/j.cellin.2022.100060

**Published:** 2022-10-11

**Authors:** Lanfang Li, Bing Zhang, Xiaomei Tang, Quntao Yu, Aodi He, Youming Lu, Xinyan Li

**Affiliations:** a Department of Physiology, School of Basic Medicine and Tongji Medical College, Huazhong University of Science and Technology, Wuhan, 4030030, China; b Institute for Brain Research, Wuhan Center of Brain Science, Huazhong University of Science and Technology, Wuhan, 430030, China; c Department of Anatomy, School of Basic Medicine and Tongji Medical College, Huazhong University of Science and Technology, Wuhan, 430030, China; d Department of Pathophysiology, School of Basic Medicine and Tongji Medical College, Huazhong University of Science and Technology, Wuhan, 430030, China

**Keywords:** Alzheimer's disease, D28K, Cholinergic neurons, Anxiety-like behavior, NRADD

## Abstract

Cholinergic neurons in the basal forebrain constitute a major source of cholinergic inputs to the forebrain, modulate diverse functions including sensory processing, memory and attention, and are vulnerable to Alzheimer's disease (AD). Recently, we classified cholinergic neurons into two distinct subpopulations; calbindin D28K-expressing (D28K^+^) versus D28K-lacking (D28K^−^) neurons. Yet, which of these two cholinergic subpopulations are selectively degenerated in AD and the molecular mechanisms underlying this selective degeneration remain unknown. Here, we reported a discovery that D28K^+^ neurons are selectively degenerated and this degeneration induces anxiety-like behaviors in the early stage of AD. Neuronal type specific deletion of NRADD effectively rescues D28K^+^ neuronal degeneration, whereas genetic introduction of exogenous NRADD causes D28K^−^ neuronal loss. This gain- and loss-of-function study reveals a subtype specific degeneration of cholinergic neurons in the disease progression of AD and hence warrants a novel molecular target for AD therapy.

## Highlights

• D28K^+^ cholinergic neurons are selectively degenerated in the early stage of AD.
• Deletion of NRADD in D28K^+^ neurons protects against neurodegeneration.
• Expression of exogenous NRADD in D28K^−^ neurons causes neurodegeneration.

## Graphical abstract

**Figure:**
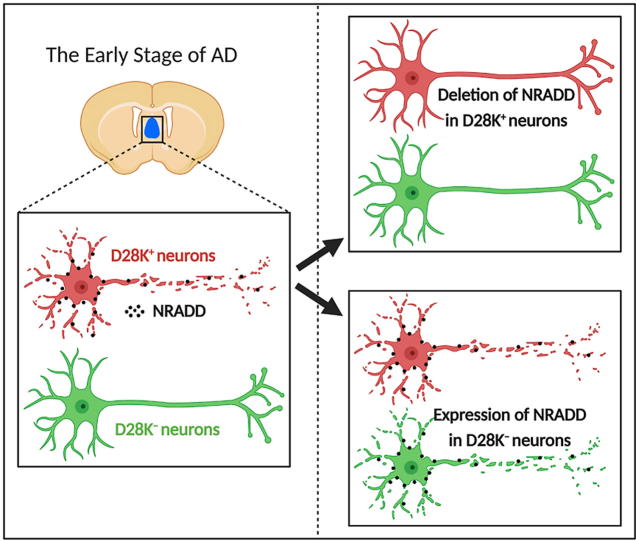

## 1 Introduction

Cholinergic neurons in the basal forebrain including the medial septum (MS) are a predominant source of cholinergic projections in the entire mammalian forebrain (Zaborszky et al., 2015; Li et al., 2018c; Wu et al., 2014), modulate physiology and behaviors in health (Furey et al., 2000; Picciotto et al., 2012; Chen et al., 2015; Jiang et al., 2016; Bentley et al., 2004; Goard and Dan, 2009; Parikh et al., 2007) and disease (Ballinger et al., 2016; Grothe et al., 2012; Hampel et al., 2018; Teipel et al., 2005). It is the most widely used pharmacological target for ameliorating the progression of Alzheimer's disease (AD), which is the leading cause of aging worldwide (Hampel et al., 2018; Hardy, 2006).

Pathological lesions and optogenetic manipulations of cholinergic neurons in the basal forebrain have shown a wide range of the behavioral functions, including learning and memory (Furey et al., 2000; Jiang et al., 2016; Knox and Keller, 2016), reward (Crouse et al., 2020; Herman et al., 2016), plasticity (Buchanan et al., 2010; Gu and Yakel, 2011; Martinello et al., 2015), attention (Parikh et al., 2007; Bentley et al., 2008; Guillem et al., 2011; Pinto et al., 2013; Sarter and Bruno, 2000), sleep-wake cycle (Xu et al., 2015) and even arousal (Motelow et al., 2015). These divergent behavioral functions have suggested that there are diverse types of cholinergic neurons in the brain. However, the nature of this diversity and its potential role in shaping the behaviors of individual cholinergic neuronal types remain unknown.

Recently, we have developed multiplexing technologies, aiming to functionally classify individual subtypes of cholinergic neurons in adult brain(Li et al., 2022). We have for the first time reported two molecularly defined subsets of cholinergic neurons in the basal forebrain of mice, macaca fascicularis and humans; calbindin D28K-expressing (D28K^+^) versus D28K-lacking (D28K^−^) cholinergic neurons (Li et al., 2022). D28K acts as Ca^2+^ buffer and sensor in mammalian cells (Lutz et al., 2003; Roberts, 1994). Specifically, we have generated a mutant strain of TERM mice, or a *t*riple *e*nzymatic *r*ecombination *m*utant strain of mice, in which CRE and FLP recombinases were expressed in D28K^+^ neurons, whereas CRE and DRE recombinases were engineered to be expressed in D28K^−^ neurons. This mutation has allowed us to manipulate two distinct subsets of cholinergic neurons and visualize them in vivo. Subsequently, we have carried out population cell RNA-seq and uncovered two distinct transcriptional profiles; *NRADD*, *Aifm3* and 30 other genes in D28K^+^ neurons versus *Gga3*, *Lrrtm4* and 21 other genes in D28K^−^ neurons. Finally, we have developed genetically modified virus synaptic tracing vectors, and by using these vectors we have characterized two distinct cholinergic subnetwork systems, playing different roles in the anxiety-like behaviors and spatial memory (Li et al., 2022).

Cholinergic neurons are selectively degenerated in the early stage of AD from both mice and human beings (Ballinger et al., 2016; Grothe et al., 2012; Hampel et al., 2018; Teipel et al., 2005). Yet, which of these two distinct subsets of cholinergic neurons are selectively degenerated and whether this selective degeneration contributes to the disease progression remain unknown. Here, we have demonstrated that D28K^+^ neurons are selectively degenerated in the early stage of an AD mouse model via NRADD transcription.

## 2 Results

### 2.1 A selective degeneration of D28K^+^ cholinergic neurons in the early stage of AD

**Fig. 1:**
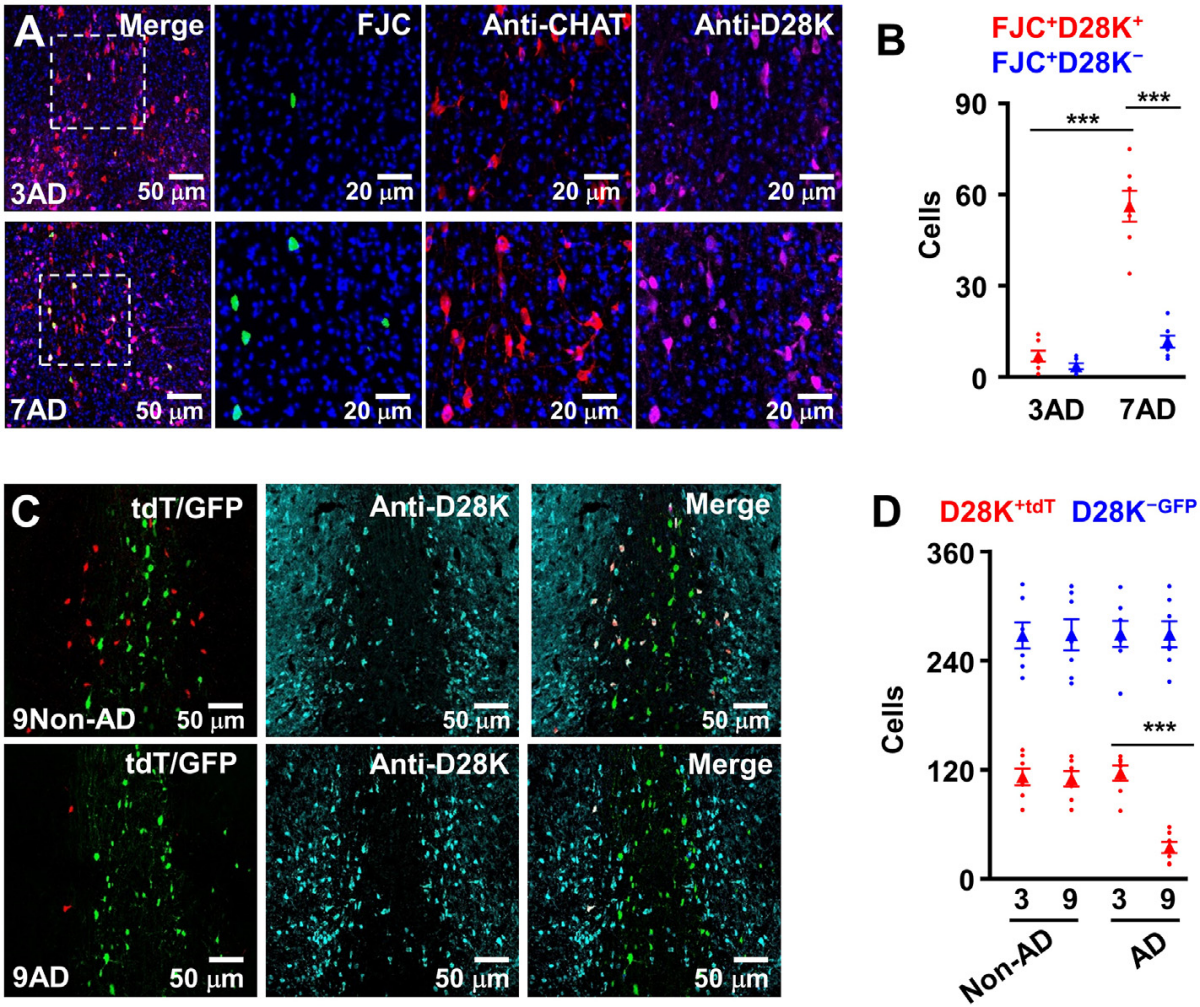
**A selective degeneration of D28K**^**+**^**neurons in early stage of AD.** (A) Representative images showing the labeling of the brain sections from male AD mice at 3 or 7 months old of age with FJC (green), anti-CHAT (red) anti-D28K (violet). (B) A selective degeneration of D28K^+^ neurons in AD mice. A plot showing the numbers of FJC-positive D28K^+^ (red) and D28K^−^ (blue) neurons in the MS from the individual (circles) AD mice at 3 or 7 months old of age and their average per group (triangles, mean ​± ​SEM, n ​= ​5 mice per group, ∗∗∗p ​< ​0.001, One-way *ANOVA*, Bonferroni’ s post hoc test). (C) A selective loss of D28K^+^ neurons in AD mice. Representative images showing D28K^+tdT^ (red) and D28K^−GFP^ (green) neurons in the MS from non-AD or AD mice at 9 months old of age. The sections were stained with anti-D28K (light blue). (D) A plot showing that numbers of D28K^+​tdT^ (red) and D28K^−GFP^ (blue) neurons in the MS from the individual (circles) non-AD/TERM (control) and AD/TERM mice at 3 or 9 months old of age and their average per group (triangles, mean ​± ​SEM, n ​= ​7 mice per group, ∗∗∗p ​< ​0.001, Non-AD versus AD in 9 months, One-way *ANOVA*, Bonferroni's post hoc test).

To determine the vulnerability of two distinct cholinergic subpopulations in the disease progression of AD, we generated AD/TERM mice by crossing TERM mice with AD mice (APP/PS1 mice in a C57BL/6 genetic background), which displayed amyloid-β peptide (Aβ) plaques and neurofibrillary tangles similar with that seen in human patients (Duyckaerts et al., 2008; Bilkei-Gorzo, 2014; Kosel et al., 2020). We then injected the rAAV2/9-hSyn–FSF–FLEX-tdTomato (tdT) together with the rAAV2/9-hSyn-RSR-FLEX-enhanced green fluorescence protein (GFP) infectious virus particles into the MS of AD/TERM mice, resulting in the expression of tdT in D28K^+^ neurons (D28K^+ ​tdT^) and GFP in D28K^−^ cholinergic neurons (D28K^−GFP^), respectively (Fig. S1). The vulnerability of D28K^+​tdT^ versus D28K^−GFP^ cholinergic neurons was then assessed. The age-matched non-AD/TERM mice were used as controls. We found that a large amount of D28K^+^ neurons in the MS were labeled with fluoro-jade C (FJC, FJC^+^), a marker for degenerated neurons (Tu et al., 2010), in AD/TERM mice at 7 months old of age (Fig. 1A and B). Consistent with FJC labeling, the numbers of D28K^+^ neurons decreased to 31.0% of the age-matched controls at 9 months old of age (Fig. 1C and D). No significantly difference of D28K^−^ neurons between AD and non-AD controls (Fig. 1C and D). This selective degeneration of D28K^+^ cholinergic neurons in AD mice is consistent with that seen in the early stage of AD patients (Geula et al., 2003; Riascos et al., 2011, 2014).

### 2.2 Development of anxiety-like behaviors in the early stage of AD

**Fig. 2:**
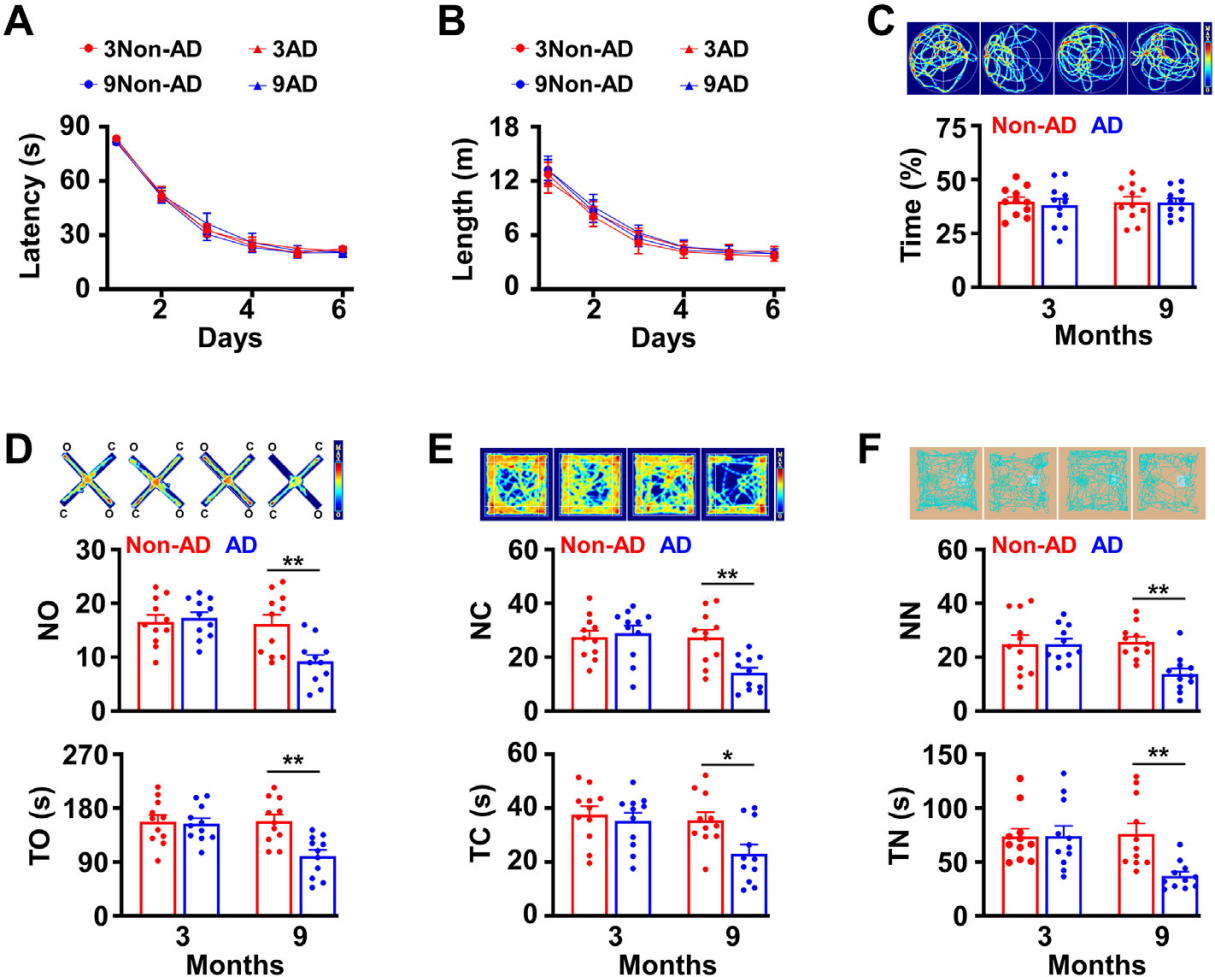
**AD mice at the early stage showanxiety-likebehaviors.** (A-C) Spatial inform processing is normal in AD/TERM mice at 9 months old of age. The latency (A) and the length (B) of swim path to reach a hidden platform and the percentage of time spent (C) in searching of a hidden platform in a targeting quadrant (quadrant 2) during the probe trial of the individual mice (circles) and their averages per group (columns, mean ± SEM, n = 11 mice per group, One-way *ANOVA*, Bonferroni's post hoc test). (D-F) AD/TERM mice at 9 months old of age show anxiety-like behaviors. Graphs show the number of entrance (NO) and time spent in open arm (TO) of the elevated plus maze (EPM, D), the number of entrance (NC) and time spent in the center (TC) of the open field (OF, E), the number of entrance (NN) and time spent exploring novel object (TN) of novel object recognition (NO, F), from the individual (circles) non-AD/TERM control (red) and AD/TERM (blue) mice at 3 and 9 months old of age and their averages per group (columns, mean ​± ​SEM, n ​= ​11 mice per group, ∗p ​< ​0.05, ∗∗p ​< ​0.01, Non-AD versus AD in 9 months, One-way *ANOVA*, Bonferroni’ s post hoc test).

Recently, we have shown that two distinct subsets of cholinergic neurons play different roles in the behaviors; D28K^+^ cholinergic neurons regulate the anxiety-like behaviors, whereas D28K^−^ neurons encode spatial memory (Li et al., 2022). Given that D28K^+^ neurons are selectively degenerated in the early stage of AD, we hypothesized that this degeneration induces the anxiety-like behaviors. To test this hypothesis, we examined the behavioral phenotypes of AD mice by using a large battery of the behavioral screening tests. We found that AD mice at 9 months old of age were normal in spatial memory encoding, based on the Morris water maze tests (Fig. 2A–C), but, they displayed a significant reduction in the number of entrance (NO) and time spent in the open arm (TO) in elevated plus maze (EPM, Fig. 2D), the number of entrance (NC) and time spent in the center (TC) in the open field (OF, Fig. 2E), and the number that the animal encounters with (NN) and time that animal explores (TN) the novel object in the novel object recognition tests (NO, Fig. 2F). This finding agrees with the previous studies showing that anxiety-like behaviors occur earlier than a loss of spatial memory in human AD patients (Pietrzak et al., 2015).

### 2.3 Deletion of NRADD protects against neurodegeneration

**Fig. 3:**
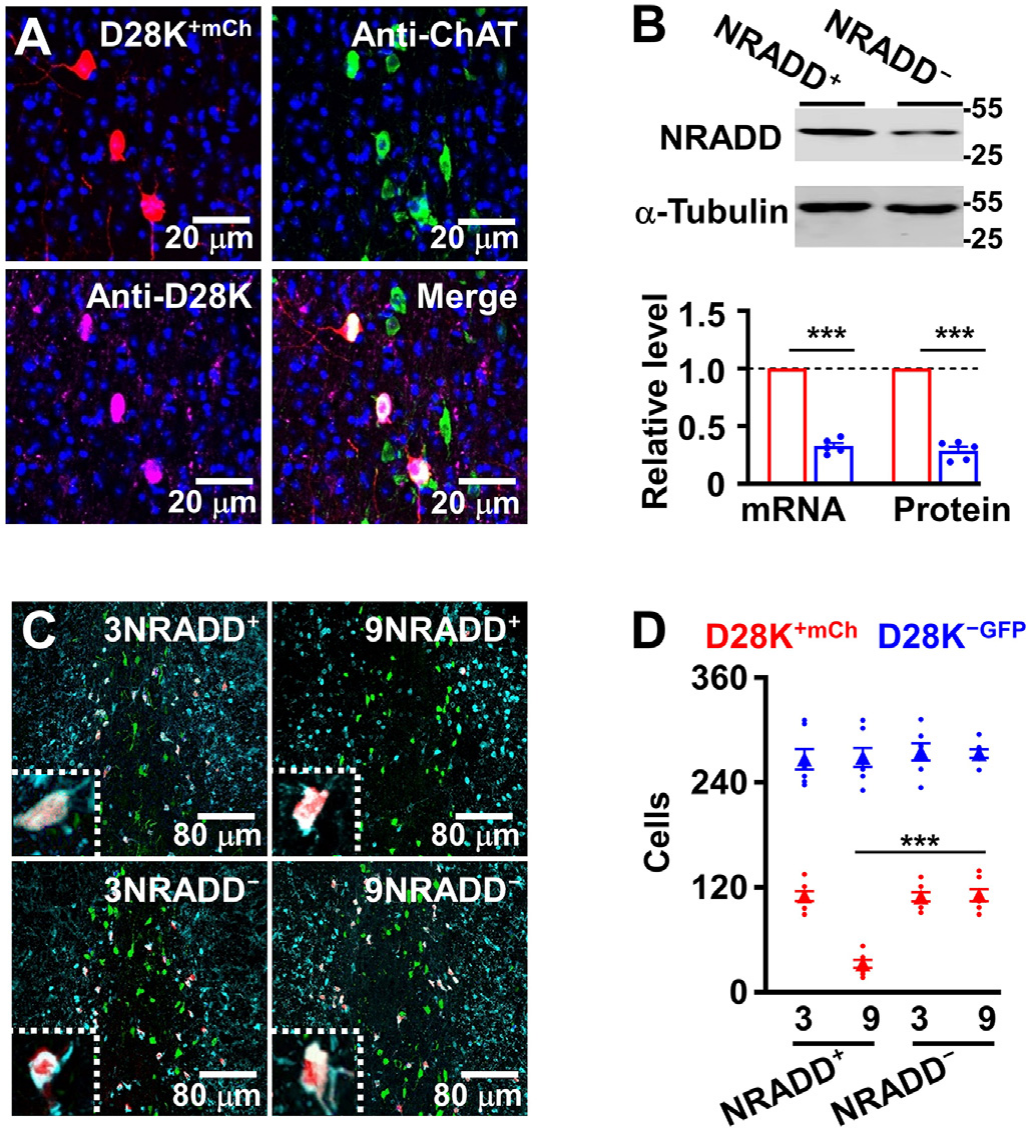
**Deletion of NRADD protects against neurodegeneration in AD.** (A) The representative image showing mCherry (mCh)-expressing neurons (red) labeled with anti-ChAT (green) and anti-D28K (violet) in the MS of AD/TERM after the injection of rAAV2/9-hSyn–FSF–FLEX-SaCas9-mCh and rAAV2/9-U6-sgRNAs-NRADD virus. (B) Knockdown of *NRADD* in D28K^+^ neurons from AD/TERM mice. Representative blots (top) of NRADD protein in D28K^+^ neurons from AD/TERM mice at 3 months old of age with (NRADD^−^) or without (NRADD^+^) *NRADD* knockdown. A plot (bottom) showing NRADD mRNA and protein levels, normalized to α-tubulin (defined as 1.0), in D28K^+^ neurons from the individual NRADD^+^ (red) and NRADD^−^ (blue) AD/TERM mice (circles) and their averages per group (columns, mean ​± ​SEM, n ​= ​5 mice per group, ∗∗∗p ​< ​0.001, unpaired *t*-tests). (C and D) *NRADD* knockdown protects D28K^+^ neurons from degeneration in AD/TERM mice. Representative images (C) showing anti-D28K (light blue) labeled D28K^+​mCh^ (red) and D28K^−GFP^ (green) cholinergic neurons in the MS of AD/TERM mice with (NRADD^−^) or without (NRADD^+^) *NRADD* knockdown at 3 or 9 months old. A plot (D) showing the numbers of D28K^+^​^mCh^ (red circles) versus D28K^−GFP^ (blue circles) cholinergic neurons in the MS from the individual NRADD^−^ and NRADD^+^ AD/TERM mice at 3 or 9 months old of age and their averages per group (triangles, mean ​± ​SEM, n ​= ​9 mice per group, ∗∗∗p ​< ​0.001, 9NRADD^+^ versus 9NRADD^−^ for D28K^+^ neurons, One-way *ANOVA*, Bonferroni's post hoc test).

**Fig. 4:**
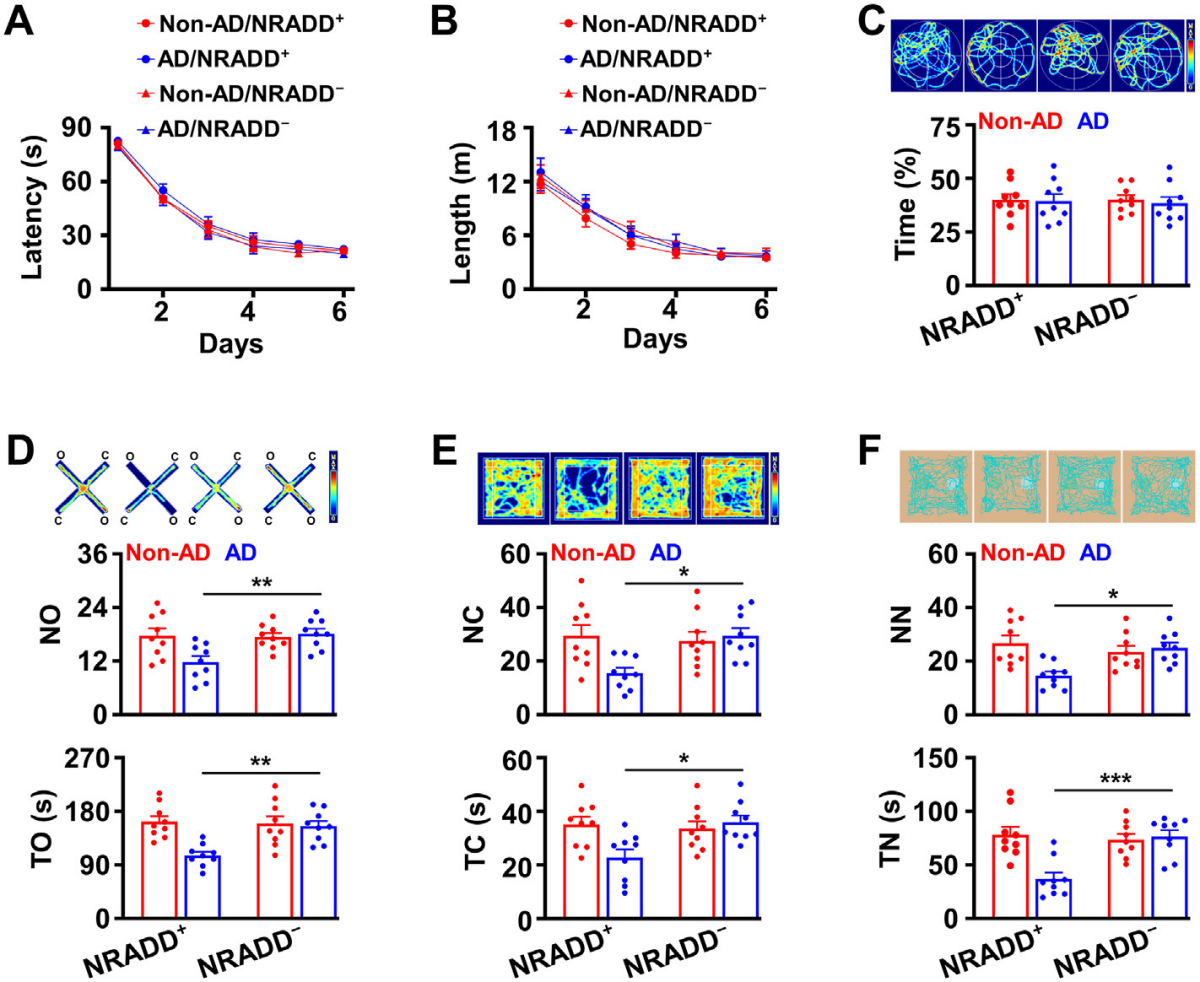
**Deletion of NRADD produces the therapeutic effects against anxiety-like behaviors in AD.** (A-C) *NRADD* knockdown produces no effects on spatial information processing. The latency (A) and the length (B) of swim path to reach a hidden platform and the percentage of time spent (C) in searching of a hidden platform in targeting quadrant (quadrant 2) during the probe trial of the individual (circles) NRADD^−^ and NRADD^+^ AD/TERM (red) and non-AD/TERM (blue) mice at 9 months old of age and their averages per group (columns, mean ± SEM, n = 11 mice per group, One-way *ANOVA*, Bonferroni's post hoc test). (D-F) *NRADD* knockdown inhibits anxiety-like behaviors in AD/TERM mice. Graphs showing the number of entrance (NO) and time spent (TO) in open arm of the elevated plus maze (EPM, D), the number of entrance (NC) and time spent (TC) in the center of the open field (OF, E), the number of entrance (NN) and time spent (TN) exploring novel object of novel object recognition (NO, F) from the individual (circles) NRADD^−^ and NRADD^+^ AD/TERM (red) and non-AD/TERM (blue) mice at 9 months old of age and their averages per group (columns, mean ​± ​SEM, n ​= ​11 mice per group, ∗p ​< ​0.05, ∗∗p ​< ​0.01, ∗∗∗p ​< ​0.001, AD/NRADD^+^ versus AD/NRADD^−^, One-way *ANOVA*, Bonferroni's post hoc test).

Recently, we have shown that two distinct subtypes of cholinergic neurons express mutually exclusive marker genes; *NRADD*, *Aifm3* and 30 other genes in D28K^+^ neurons versus *Gga3*, *Lrrtm4* and 21 other genes in D28K^−^ neurons (Li et al., 2022). Subsequently, we wanted to determine which of these genes that were specifically expressed in D28K^+^ neurons mediate neurodegeneration in the early stage of AD. We developed rAAV2/9 virus-mediated CRISPR-Cas9 pooled gene knockdown in vivo. Specifically, we injected rAAV2/9-hSyn–FSF–FLEX-SaCas9-mCherry virus, mixed with rAAV2/9-U6-sgRNAs infectious virus particles into the MS of AD/TERM mice by targeting three groups of the genes enriched in D28K^+^ neurons. These included *kcnh1*, *kcnu1*, *kctd15*, *casp4*, *aifm3*, *NRADD*, *bcl2l12*, *slc25a18* and *D28K*. 14 days after the injection, D28K^+^ neurons were purified and mRNAs of the targeted genes were analyzed (Fig. S2). We found that knockdown of *n*eurotrophin *r*eceptor *a*like *d*eath *d*omain protein (NRADD, Fig. 3A and B), in which the transmembrane and cytoplasmic regions are highly homologous to death receptor, such as p75 (Nadezhdin et al., 2019; Wang et al., 2003), effectively protected D28K^+^ cholinergic neurons from degeneration (Fig. 3C and D and Fig. S3) and completely eliminated the anxiety-like behaviors at the early stage of AD mice, without altering the performance in the Morris water maze tests (Fig. 4A–F).

### 2.4 Expression of NRADD causes neurodegeneration

**Fig. 5:**
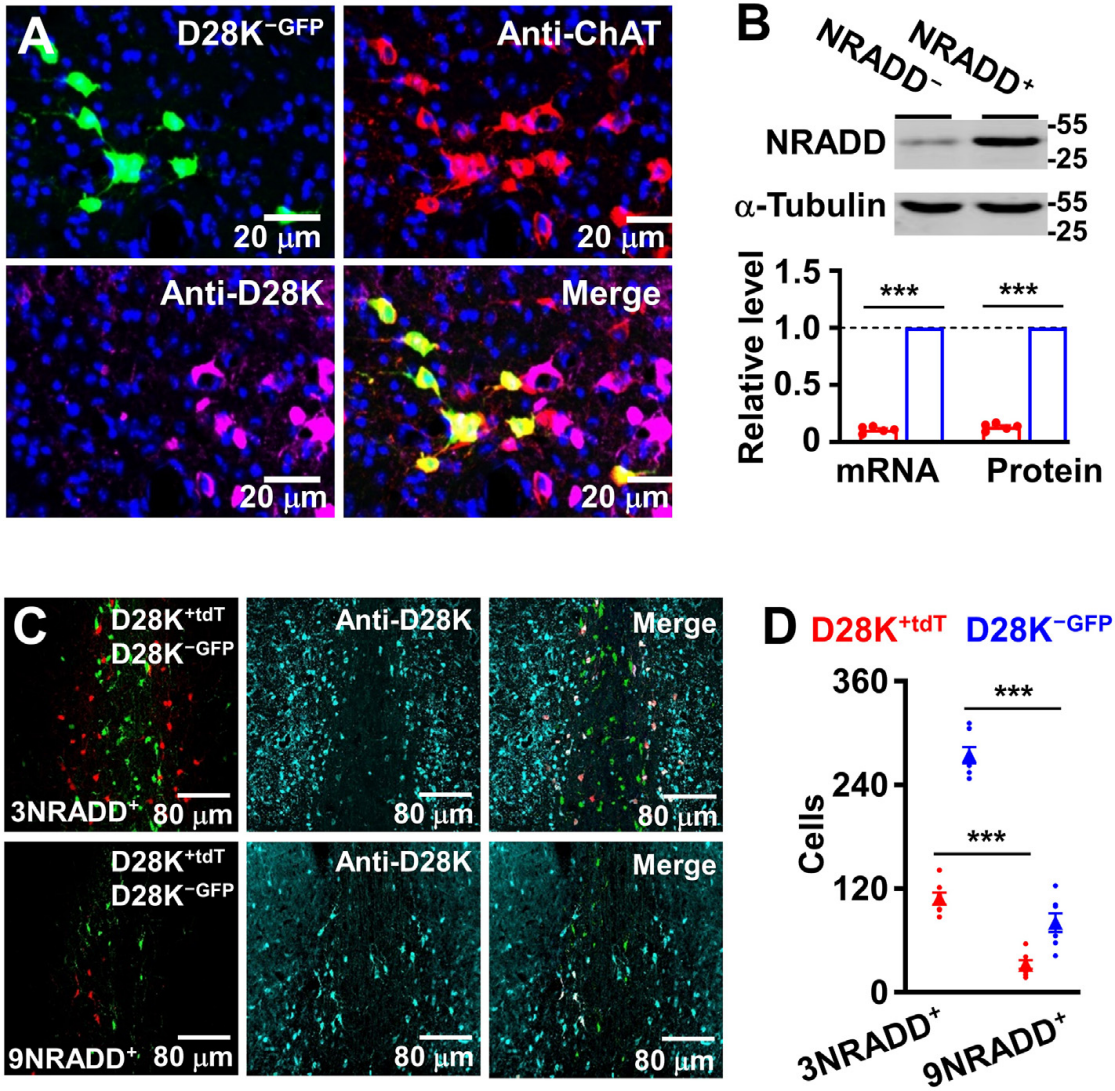
Expression of exogenous NRADD degenerates D28K^−^ neurons in AD. (A) The representative image showing GFP-expressing neurons (green) labeled with anti-ChAT (red), but not with anti-D28K (violet) in the MS of AD/TERM after the injection of rAAV2/9-hSyn-RSR-FLEX-NRADD-GFP virus. (B) Expression of exogenous NRADD in D28K^−^ neuron. Representative blots (top) of NRADD protein in D28K^−^ neurons expressing exogenous NRADD-GFP (D28K^−NRADD−GFP^) or GFP (D28K^−GFP^) from AD/TERM mice at 3 months old of age and a plot showing NRADD mRNA and protein levels, as normalized to α-tubulin (defined as 1.0) in D28K^−GFP^ (red) and D28K^−NRADD−GFP^ (blue) neurons from the individual AD/TERM mice (circles) and their averages per group (columns, mean ​± ​SEM, n ​= ​5 mice per group, ∗∗∗p ​< ​0.001, unpaired *t*-tests). (C and D) Expression of exogenous NRADD degenerates D28K^−^ neurons in AD/TERM mice. Representative images (C) and the numbers (D) of D28K^+ ​tdT^ (red circles) and D28K^−NRADD−GFP^ (blue circles) neurons in the MS from the individual AD/TERM mice at 3 or 9 months old of age and their averages per group (triangles, mean ​± ​SEM, n ​= ​9 mice per group, ∗∗∗p ​< ​0.001, unpaired *t*-tests).

**Fig. 6:**
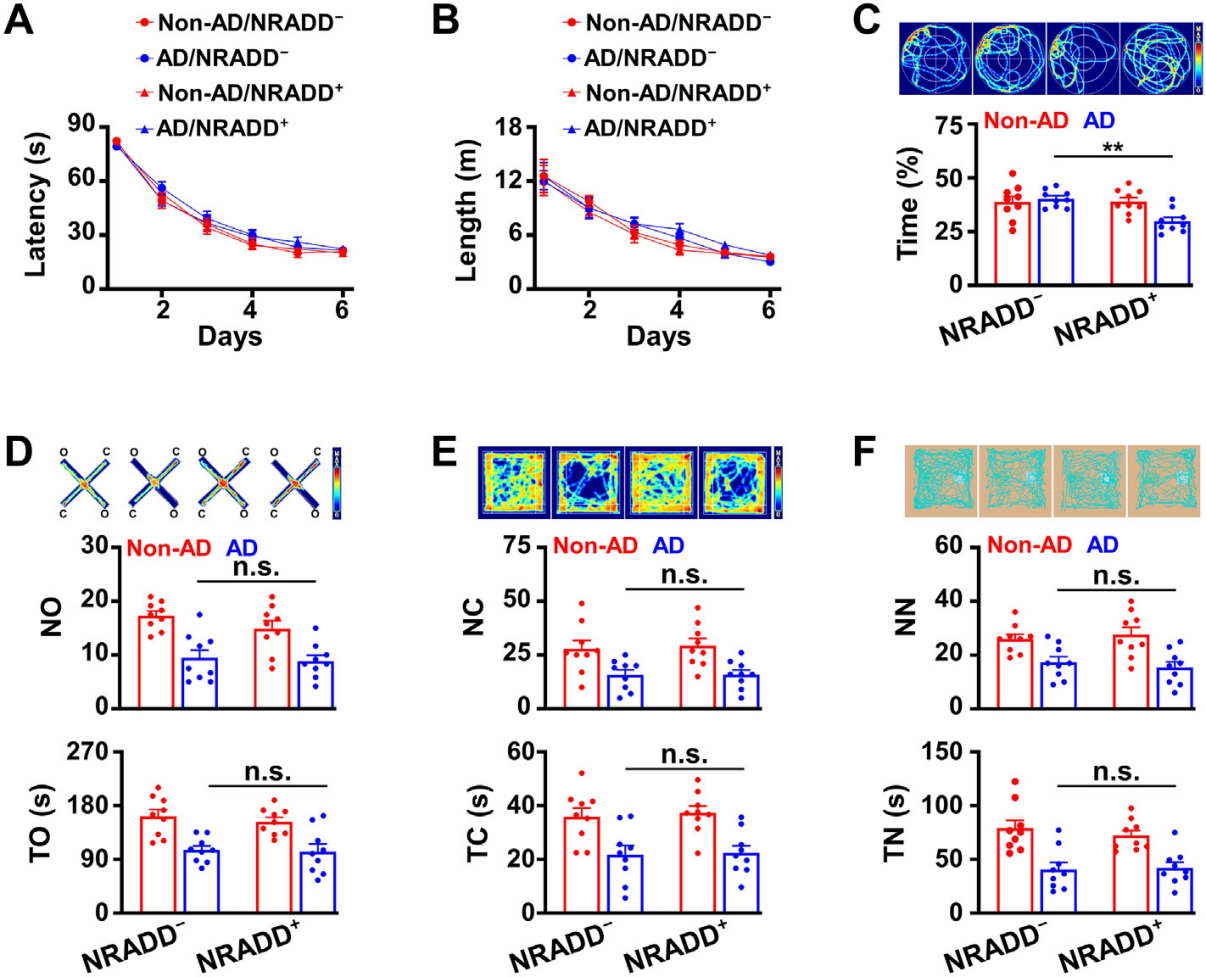
**Expression of exogenous NRADD in D28K**^**−**^**neurons impairs spatial memory.** (A-C) Spatial memory is impaired by expressing exogenous NRADD in D28K^−^ neurons. The latency (A) and length (B) of swim path to reach a hidden platform and the percentage of time spent (C) in searching of a hidden platform in targeting quadrant (quadrant 2) during the probe trial of the individual (circles) D28K^−NRADD−GFP^ and D28K^−GFP^ non-AD/TERM (blue) and AD/TERM (red) mice at 9 months old of age and their averages per group (columns, mean ​± ​SEM, n ​= ​11 mice per group, ∗∗p ​< ​0.01, One-way *ANOVA*, AD/NRADD^−^ versus AD/NRADD^+^, Bonferroni's post hoc test). (D-F) Presence of anxiety-like behaviors in D28K^−NRADD−GFP^ AD mice. Graphs show NO and TO of the elevated plus maze (EPM, D), NC and TC of the open field (OF, E), NN and TN of the novel object recognition (NO, F), from the individual (circles) D28K^−NRADD−GFP^ versus D28K^−GFP^ non-AD/TERM (blue) and AD/TERM (red) mice at 9 months old of age and their averages per group (columns, mean ± SEM, n = 11 mice per group, p > 0.1, AD/NRADD^−^ versus AD/NRADD^+^ One-way *ANOVA*, Bonferroni's post hoc test).

To further determine a role of NRADD in a selective degeneration of cholinergic neurons, we expressed exogenous NRADD in D28K^−^ neurons of AD mice (Fig. 5A and B). This expression caused a robust loss of D28K^−^ neurons (Fig. 5C and D), impaired spatial memory (Fig. 6A–C). This impairment of spatial memory was associated with anxiety-like behaviors (Fig. 6D–F). Together, these data demonstrated that NRADD fulfilled both necessary and sufficiency conditions as novel mediator for a selective degeneration of cholinergic neurons in AD and hence can be considered as therapeutic target for the early intervention of the disease progression.

## 3 Discussion

Cholinergic neurons in the basal forebrain are the major sources of cholinergic projections to neocortex and hippocampus (Bloem et al., 2014; Li et al., 2018c; Zaborszky et al., 2015) and provide the predominant cholinergic network systems directly engaged in learning and memory, as well as in emotion (Bentley et al., 2004; Bloem et al., 2014; Chen et al., 2015; Furey et al., 2000; Goard and Dan, 2009; Jiang et al., 2016; Li et al., 2018c; Parikh et al., 2007; Picciotto et al., 2012; Zaborszky et al., 2015). Recently. we have identified two distinct subtypes (D28K^+^ versus D28K^−^) of cholinergic neurons in the basal forebrain of adult mice; D28K^+^ neurons control the induction of the anxiety-like behaviors, whereas D28K^−^ neurons encode spatial memory (Li et al., 2022). In the present study, we have uncovered that D28K^+^ neurons are selectively degenerated and this degeneration mediates early onset of the anxiety-like behaviors in AD via NRADD.

Cholinergic neurons are selectively degenerated in the early stage of AD from both mice and human beings (Ballinger et al., 2016; Grothe et al., 2012; Hampel et al., 2018; Teipel et al., 2005). The molecular mechanisms underlying cholinergic neurodegeneration have mainly focused on amyloid-β senile plague and neurofibrillary tau tangles (Li et al., 2018a; Robinson et al., 2017). But, the most strategies by targeting these molecular mechanisms for the therapeutic interventions of the disease progression of AD have failed in clinic trials (Khan et al., 2020; Mangialasche et al., 2010). In our present study, we have revealed that D28K^+^, but not D28K^−^ cholinergic neurons are degenerated at the early stage of AD mice. We have determined the functional significance of D28K^+^-enriched genes in this selective neurodegeneration and found that deletion of NRADD survived D28K^+^ neurons from the disease, whereas engineered D28K^−^ neurons with the expression of exogenous NRADD causes D28K^−^ cholinergic neurons to be degenerated.

NRADD death domain transmembrane protein that contains two folded regions-α-helical transmembrane domain and globular C-terminal death domain (Nadezhdin et al., 2019). N-terminally truncated NRADD is processed by γ-secretase following binding with a precursor protein of nerve growth factor (pro-NGF) (Volosin et al., 2006) and has the same susceptibility to γ-secretase inhibitors as the secretion of amyloid β (Gowrishankar et al., 2004). Accordingly, this study has provided a novel mechanism for a selective degeneration of cholinergic neurons in AD, possibly by a proteolytic process of NRADD in D28K^+^ neurons (Fig. S4) and hence warrants a promising molecular target for AD therapy.

## 4 Materials and methods

### 4.1 Animals and virus

In this study, we used male mice to avoid the potential differences between genders. Mice were bred and reared under the same conditions in accordance with institutional guidelines and the Animal Care and Use Committee of the animal core facility at Huazhong University of Science and Technology, Wuhan, China and housed in groups of three to five mice/cage under a 12-h light-dark cycle, with lights on at 8:00 a.m., at a consistent ambient temperature (21 ± 1 °C) and humidity (50 ± 5%). We performed all behavioral tests during the light phase of the cycle, as described before(He et al., 2022; Jing et al., 2021; Li et al., 2018b).

To selectively target in D28K^+^ and D28K^−^ cholinergic neurons in AD mice, we generated AD/TERM mice, in which CRE and FLP recombinase were expressed in D28K^+^ neurons, whereas CRE and DRE recombinase were expressed in D28K^−^ neurons, as described before (Li et al., 2022), and also shown in Fig. S1A. Age-matched non-AD/TERM mice were used as controls.

AD amyloid disease model mice (APP/PS1 mice, or AD mice) with C57BL/6 genetic background were purchased from the Jackson Laboratory (Stock No: 034829), which are double transgenic mice expressing a chimeric mouse/human amyloid precursor protein (Mo/HuAPP695swe) and a mutant human presenilin 1 (PS1-dE9), as described previously (Jankowsky et al., 2004). Heterozygous AD mice were used in all experiments. ChAT-CRE mice were purchased from Jackson Laboratory (Stock No: 006410). D28K-FLP mice, in which FLP was expressed under the control of the D28K promoter, were generated by Gem-Pharma-Tech Co Ltd, Nanjing, China. The C_on_/F_off_-DRE mice with the ROSA26-pCAG-Lox*P*-STOP-Lox*P*-FRT-DRE-FRT-WPRE-pA construct were generated by Shanghai Model Organisms Center, Inc., Shanghai, China. To determine the specificity of CRE-FLP and CRE-DRE recombination in D28K^+^ versus D28K^−^ cholinergic neurons, we assessed whether CRE-FLP activates the reporter expression only in D28K^+^ neurons, whereas CRE-DRE activates the reporter expression only in D28K^−^ neurons. We injected a high titer (0.1 μl, 8 × 10^12^ genomic particles/ml) of the rAAV2/9-hSyn–FSF–FLEX-tdT together with the rAAV2/9-hSyn-RSR-FLEX-GFP virus (0.1 μl, 8 × 10^12^ genomic particles/ml) into the MS of TERM mice. The coordinates of the stereotaxic virus injections were AP: 0.8 mm, ML: 0 mm, and DV: 4.5 mm from bregma. This injection resulted in the expression of tdT in D28K^+^ neurons and GFP in D28K^−^ neurons only (Figs. S1B–C).

### 4.2 CRISPR-Cas9 genetic function screening

To screen the function of the genes in D28K^+^ neurons, we designed five sgRNAs that specifically targets to each enriched gene. We used five sgRNAs that specifically targets to zfy2 (sgRNA-zfy2) as a control. Zfy2 was used as it was not expressed in any types of neurons in the brain. We expressed sgRNAs in D28K^+^ neurons by the injection of a pooled virus particle; the rAAV2/9-hSyn–FSF–FLEX-SaCas9-mCh together with the rAAV2/9-U6-sgRNAs virus into the MS of AD/TERM mice at 3 months old of age, resulting in the expression of mCh and sgRNAs in D28K^+^ neurons (D28K^+​mCh^). qPCR was used for in vivo knockdown validation (Fig. S2).

To knockdown *NRADD* in D28K^+^ neurons, we used five sgRNAs that specifically target to NRADD (sgRNAs-NRADD). Five sgRNAs that specifically targets to zfy2 (sgRNA-zfy2) as a control. We expressed sgRNA-NRADD or sgRNA-zfy2 in D28K^+^ neurons by the injection of a pooled virus particle; the rAAV2/9-hSyn–FSF–FLEX-SaCas9-mCh together with the rAAV2/9-hSyn-RSR-FLEX-GFP and the rAAV2/9-U6-sgRNAs-NRADD virus or the rAAV2/9-U6-sgRNAs-zfy2 virus into the MS of AD/TERM mice at 3 months old of age, resulting in the expression of mCh and sgRNAs-NRADD or sgRNAs-zfy2 in D28K^+^ neurons (D28K^+mCh^) and GFP in D28K^−^ neurons (D28K^−GFP^). Western blots and qPCR were used for in vivo knockdown validation. 12 days (3 months old of age) or 6 months (9 months old of age) after the injection, the numbers of D28K^+​tdT^ neurons expressing sgRNAs-NRADD (NRADD^−^) or sgRNAs-zfy2 (NRADD^+^) were then imaged and counted. The sgRNAs and primers are respectively listed in Table S1 and S2.

### 4.3 Virus

For expression of exogenous NRADD in D28K^−^ neurons, the rAAV2/9-hSyn-RSR-FLEX-NRADD-IRES-GFP was injected into the MS of AD/TERM mice. The rAAV2/9-hSyn-RSR-FLEX-GFP virus was used as a control. All the rAAVs used in this study were synthesized by Shanghai Taitool Bioscience based on a customized service.

### 4.4 Western blots

We isolated D28K^+^ neurons from the MS of AD/TERM mice after *NRADD* knockdown or expression. In brief, the brain slices were prepared and digested in buffer that contained 10 mM Tris-Cl (pH 7.6), 50 mM NaF, 1 mM Na_3_VO_4_, 1 mM EDTA, 1 mM benzamidine, 1 mM PMSF, 1 mg/10 ml papain, and a mixture of aprotinin, leupeptin, and pepstatin-A (10 μg/ml each) for 30 min. Suspended D28K^+​tdT^ and D28K^−GFP^ neurons were automatically isolated using an S3e Cell Sorter (Bio-Rad), homogenized, and diluted with a buffer that contained 200 mM Tris-Cl (pH 7.6), 8% SDS, and 40% glycerol. The protein concentration was determined using a BCA kit (Pierce, Rockford, IL). Final concentrations of 10% β-mercaptoethanol and 0.05% bromophenol blue were added, and the samples were boiled for 10 min in a water bath. The proteins in the extracts were separated by 10% SDS-PAGE and transferred to nitrocellulose membranes. The blots were scanned using an Infrared Imaging System (Odyssey, LI-COR). The blots were incubated with rabbit anti-NRADD (ABclonal Technology Co.,Ltd., Wuhan, China, generated based on a customer service). A cytoplasmic topological domain consisting of 152 amino acids (77–228) of NRADD was used as an antigen and mouse anti-α-tubulin (1:2000, Abcam, ab7291) was used as internal reference. The band densities were quantitatively analyzed using Kodak Digital Science 1D software (Eastman Kodak, New Haven, CT), as described before (Li et al., 2021a).

### 4.5 Anxiety-like behaviors

We tested mice sequentially in the EPM, OF and NO, each lasting 10 min. For the EPM tests, we used a standard mouse EPM sized maze (50 cm height of maze from floor, 63 cm full length of each arm type, 6 cm arm width, 15 cm tall closed arms, with 0.5 cm tall/wide ledges on the open arms). We placed mice in the center region of the maze at start of assay while recording behavior with a digital camera, and analyzed with TopScan tracking software (Noldus, Holland). We analyzed the parameters; including the number of entries and time spent in open arm. For the OF tests, 40 × 40 × 30 cm boxes were used. We placed mice into a corner at start of assay. We measured parameters of the number of entries into and time spent in a 20 × 20 cm square region in the center. Total distance moved and time spent immobile were also recorded. Average velocity in the open field was calculated by dividing total distance moved by time spent mobile. For the NO tests, we placed two square pyramids (objects, 10 × 10 cm of the square, 10 cm-tall) in the center of the open field box (40 × 40 × 30 cm). In the familiarization phase, a mouse was allowed to freely explore two identical objects in the same arena before being returned to the home cage for 2 h. In the test phase, this mouse was placed back to the same arena and allowed to freely explore two objects for 10 min. In this test phase, one square pyramid (the familiar object) was replaced by a novel cone (novel object, 10 cm-diameter, 10 cm-tall) at the same location. Behavior was video recorded and the time exploring each object was manually analyzed. The arena was cleaned with 70% ethanol solution after each session. The experimenters coded all animals from the experiments before quantitative analysis. Quantification was performed by the other experimenters who were unaware of the experimental conditions and treatments.

### 4.6 Morris water maze

We filled a 1.5 m-diameter swimming pool with white and non-toxic ink water. Pool temperature was maintained at 25 °C. We placed a mouse to the behavior room where this mouse was housed for the training for 1–2 days before training session, as described before (Zhu et al., 2017). The training session lasted for 6 days. In the first day of training, a mouse was allowed resting on the platform for 30 s and to have 90 s for finding the hidden platform. In case that a mouse did not find the platform within 90 s, we guided this mouse to find and stay the platform for 30 s. Throughout the period of training session, the animal was required to perform a total of 4 trials, in which a mouse was released at four different randomized release points of the pool. Immediately, after the 6-day training session, this mouse was required to perform a one-probe trial. In both training and probe trials, the behavioral tests were performed by an experimenter who was unaware of the genotypes and treatments.

### 4.7 Immunohistochemistry

Mice were sacrificed by intraperitoneally injection of an overdose of chloral hydrate and were transcardially perfused with 100 mL saline (0.9% w/v NaCl), followed by 4% Paraformaldehyde (PFA). Brains were removed and post-fixed in 4% PFA. 30 μm sagittal or coronal sections were sliced (Leica Microsystems, Wetzlar, Germany). Immunohistochemistry was performed on free-floating brain sections as described previously(Li et al., 2021b; Yang et al., 2018). In brief, staining was performed on 30 μm free-floating coronal sections and blocked in 3% normal donkey serum (room temperature for 1 h). The sections were then incubated in 50 mM Tris-HCl buffer containing 3% goat serum and 0.3% Triton X-100 with one of the following primary antibodies: mouse anti-D28K (1: 1000, Swant, 300), goat anti-ChAT (1:2000, Millipore, AB144P), for 24 h. Sections were rinsed with reacted with Tris-HCl buffer containing 3% goat serum and 0.3% Triton X-100 and reacted with conjugate-adsorbed Alexa Fluor secondary antibodies (Invitrogen) at room temperature for 1 h. Sections were rinsed, dried, and cover-slipped with fluorescence mounting medium. Fluoro-Jade C staining for degenerative neurons was performed following the instructions of the manufacturer (Biosensis, CAS#: TR-100-FJ), as described previously (Tu et al., 2010). Single or double labeling was viewed and imaged with a confocal laser-scanning microscope (Zeiss LSM800 Examiner Z1) and analyzed with a three-dimensional constructor (Image-Pro Plus software). A confocal series of images were taken at 0.5 μm intervals through the region of interest, and optical stacks of 6–12 images were produced for the figures and numbered by the experimenters. The other experimenters who were unaware of the experimental conditions counted cells from each section of the MS (0.5–1.2 mm before the bregma; reference to the mouse brain atlas, Paxinos & Franklin, 2019), and the total numbers of the labeled cells were summarized and plotted.

### 4.8 Statistical analysis

All values in the text and figure legends are represented as the mean±SEM. Unpaired two-tailed Student's *t* tests (*t*-test) and Bonferroni's post hoc test following the one-way and two-way analyses of variance (BF *ANOVA*) were used when assumptions of normality and equal variance (*F* test) were met. Significance was accepted for *p* < 0.05. Power calculations were performed using GraphPad Prism v9.0. All statistical data are summarized in Table S3.

## Data availability

Any additional information required is available from the corresponding author on reasonable request.

## Author contributions

YL and XL conceived and designed the studies and wrote the paper. LL, BZ, XT, QY and AH carried out the experiments including cell typing, synaptic tracing, CRISPR-Cas9, gene targeting, pc-RNA-seq, virus constructions and behavioral tests. All authors contributed to the data analysis and presentation in the paper.

## Declaration of competing interest

The authors declare that they have no known competing financial interests or personal relationships that could have appeared to influence the work reported in this paper.
