# Supplementary material for: A selective degeneration of cholinergic neurons mediated by NRADD in an Alzheimer's disease mouse model
Source: Cell Insight. 2022 Oct 11;1(6):100060. doi: 10.1016/j.cellin.2022.100060 (PMC10120297; doi:10.1016/j.cellin.2022.100060)
Supplement: Multimedia component 1 [file mmc1.pdf]

Fig. S1. Genetically targeting D28K^{+} and D28K^{-} neurons in AD mice

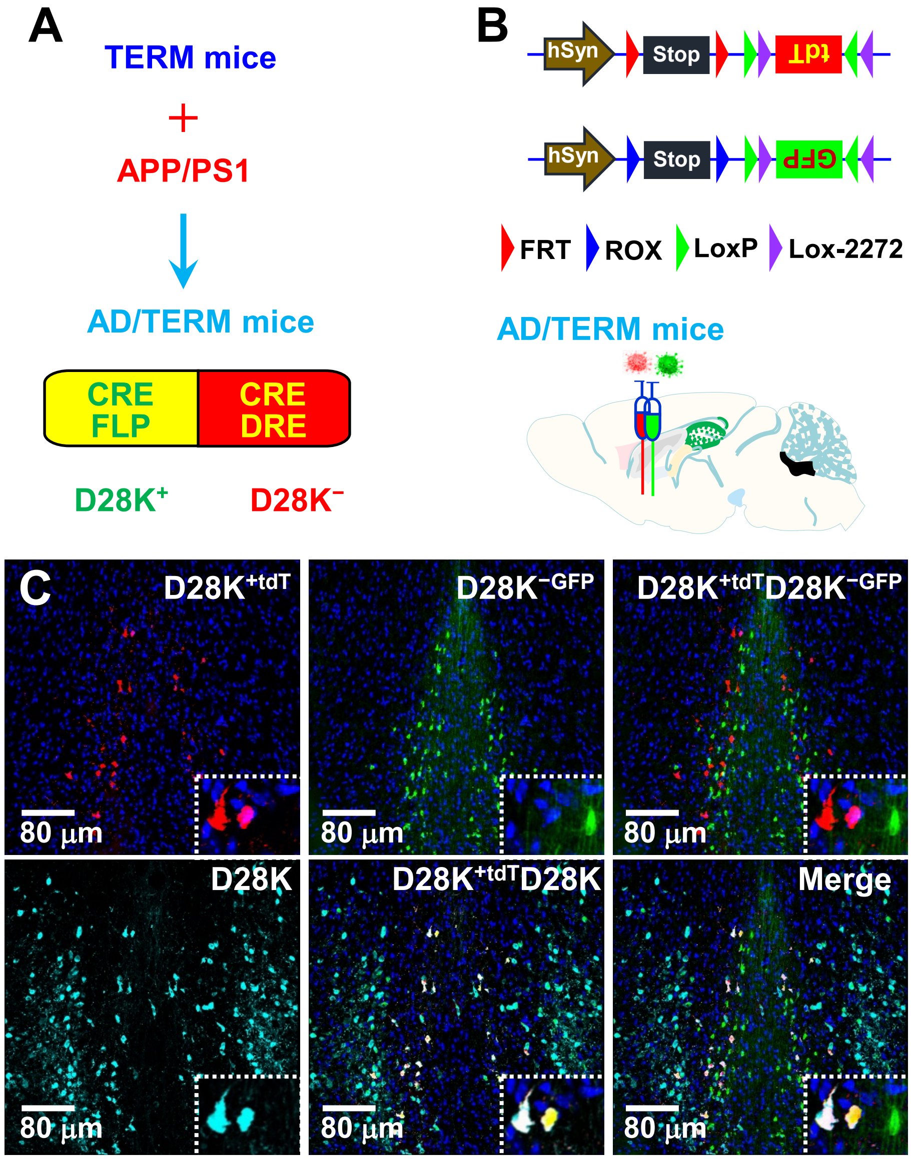

Fig. S1. Genetically targeting D28K^{+} and D28K^{-} neurons in AD mice.

(**A**) Crossing TERM mice, in which CRE was expressed in all ChAT^{+} neurons, whereas FLP was expressed in D28K^{+} ChAT^{+} neurons and DRE was expressed in D28K^{-} ChAT^{+} neurons with AD mice, resulting in the AD/TERM mice. (**B**) Genetically targeting D28K^{+} and D28K^{-} neurons selectively in AD/TERM mice by injecting FLP- and CRE-recombination-dependent or DRE- and CRE-recombination-dependent AAV virus. (**C**) Representative images showing the expression of tdT in D28K^{+} neurons (red) and GFP in D28K^{-} neurons (green) 12 days after the injection of the rAAV2/9-hSyn-FSF-FLEX-tdT together with the rAAV2/9-hSyn-RSR-FLEX-GFP virus into the MS of AD/TERM mice. Staining the sections with anti-D28K (light blue) showing D28K labeling of D28K^{+} but not D28K^{-} neurons.

Fig. S2. CRISPR-Cas9 in vivo Genetic Functional Screen

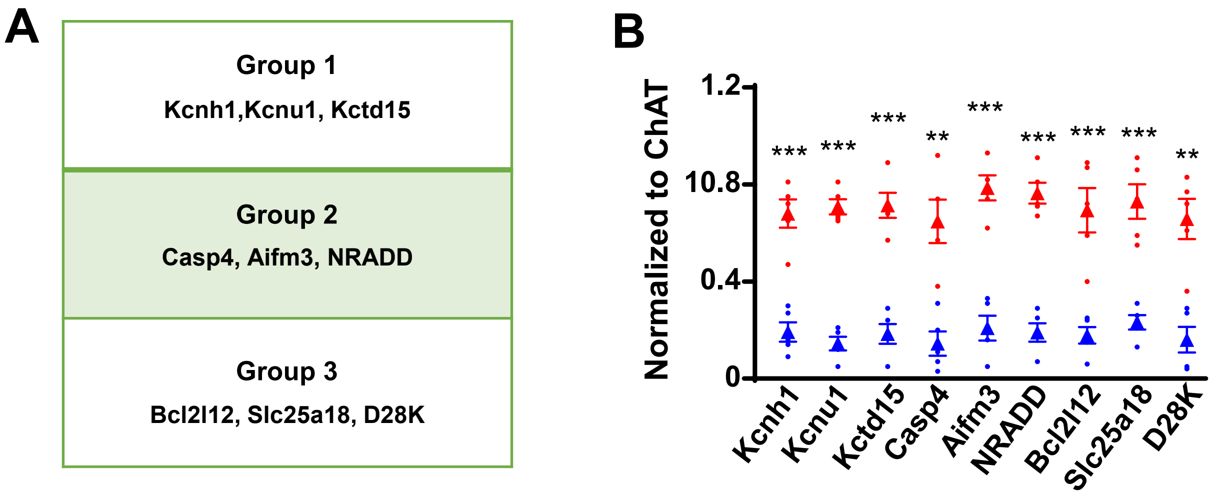

Fig. S2. CRISPR-Cas9 in vivo Genetic Functional Screen .

(**A**). The rAAV2/9-mediated CRISPR-Cas9 genetic functional screen. A list of $D28K^+$ (groups 1-3) enriched genes for knock out. (**B**) The expression levels of $D28K^+$ enriched genes before (red) and after (blue) CRISPR-Cas9 knock out in $D28K^+$ neurons from the individual mice (circles) and their averages per group (mean ± SEM, n = 5 mice per group, unpaired *t*-test). In this study, we injected the rAAV2/9-U6-sgRNAs virus together with the rAAV2/9-hSyn-FSF-FLEX-SaCas9-mCh into the MS of AD/TERM mice at 3 months old of age. 12 days after the injection, the cellular RNA were prepared from the purified $D28K^+$ neurons expressing mCh ($D28K^{+mCh}$and analyzed by qPCR (mean ± SEM, n = 5 per group, unpaired *t*-test, ***$p < 0.001$).

Fig. S3. NRADD knockdown protects D28K⁺ neurons from degeneration in AD/TERM mice

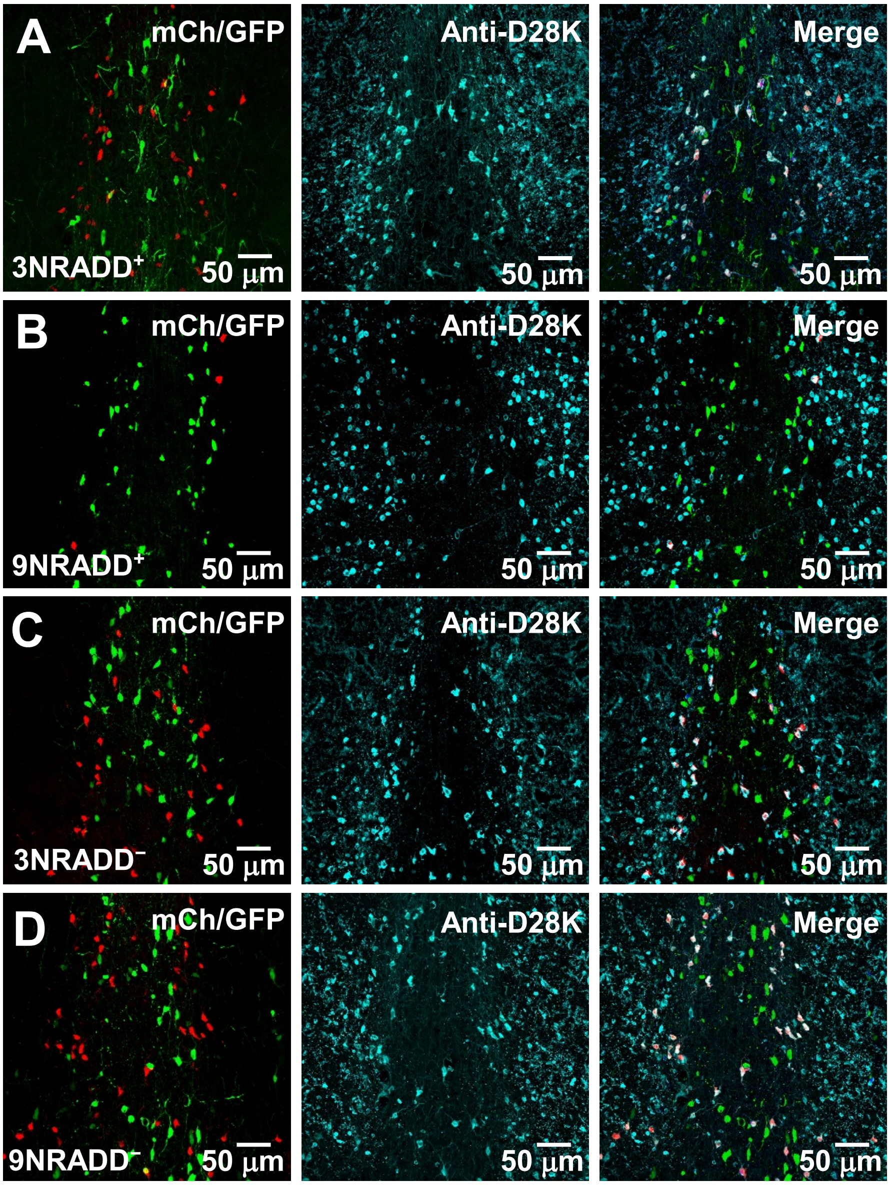

Fig. S3. NRADD knockdown protects D28K⁺ neurons from degeneration in AD/TERM mice. (**A-D**) Representative images from **Fig. 3C** showing the expression of SaCas9, mCh, and sgRNA-zyf2 in D28K⁺ (red, NRADD⁺, **A, B**) or sgRNAs-NRADD and SpCas9-mCh (red, NRADD⁻, **C, D**) and GFP in D28K⁻ (green, D28K^{-GFP}) neurons 12 days (3 months old of age) or 6 months (9 months old of age) after the injection of the rAAV2/9-hSyn-FSF-FLEX-SaCas9-mCh, the rAAV2/9-hSyn-RSR-FLEX-GFP, together with the rAAV2/9-U6-sgRNAs-zyf2 virus (**A, B**) or the rAAV2/9-U6-sgRNA-NRADDs virus (**C, D**) into the MS of AD/TERM mice at 3 months old of age. Brain sections were stained with anti-D28K (light blue) and imaged.

Fig. S4. A novel mechanism for a selective neurodegeneration via NRADD

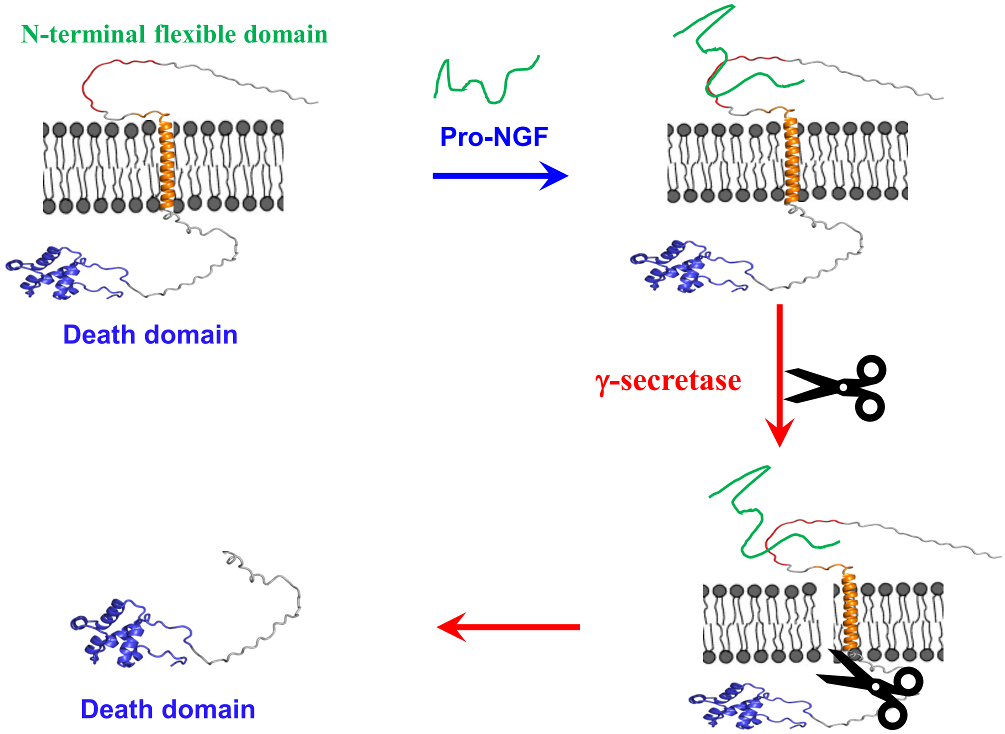

Fig. S4. A novel mechanism for a selective neurodegeneration via NRADD.

A full-length structure of NRADD was proposed based on experimental NMR data from PDB ID 6HJ7, Alpha fold and homology prediction. The death domain in blue was resolved by NMR. The loops in grey were flexible, as suggested by both NMR and Alpha fold. The transmembrane domain (TM) in orange was predicted by Alpha fold and homology with a tumor necrosis factor receptor superfamily membrane-16 or p75 neurotrophin receptor ($P75^{NTR}$). We have proposed a precursor protein of nerve growth factor (pro-NGF) binding region in red and γ-secretase cleavage site, based on homology with $P75^{NTR}$. An increase of pro-NGF in the brain of AD patients (Volosin et al., 2006), binds to N-terminal flexible domain of NRADD and allows γ-secretase into a cleavage site of NRADD. NRADD cleavage results in the production of death domain and in turn causes apoptosis of $D28K^{+}$ neurons. Thus, NRADD is a novel substrate of γ-secretase and can be considered as a therapeutic target for AD therapy.
